# Group A Streptococcal Virulence Factors and Vaccine Development—An Update

**DOI:** 10.3390/microorganisms14020357

**Published:** 2026-02-03

**Authors:** Shunyi Fan, Catherine Jia-Yun Tsai, Jacelyn Mei San Loh, Thomas Proft

**Affiliations:** 1 Department of Molecular Medicine, School of Medical Sciences, The University of Auckland, Auckland 1010, New Zealand; shunyi.fan@auckland.ac.nz (S.F.); j.tsai@auckland.ac.nz (C.J.-Y.T.); 2 Maurice Wilkins Centre for Molecular Biodiscovery, Auckland 1010, New Zealand

**Keywords:** Group A Streptococcus, *Streptococcus pyogenes*, virulence factors, vaccines

## Abstract

A Group A Streptococcus (GAS, *Streptococcus pyogenes*) is an exclusively human pathogen whose virulence is driven by a diverse array of surface structures, secreted toxins, and immune evasion mechanisms. Central to its pathogenicity is the M protein, a surface-anchored molecule that inhibits phagocytosis by interfering with complement deposition and binding host factors such as fibrinogen. GAS also secretes a wide range of toxins and enzymes that damage tissues and disrupt host defences. Streptolysin O and streptolysin S are potent cytolysins that lyse immune cells and contribute to tissue necrosis. Pyrogenic exotoxins (such as SpeA and SpeC) act as superantigens, triggering massive, dysregulated T cell activation and cytokine release, an underlying mechanism in streptococcal toxic shock syndrome. Additional factors like DNases and streptokinase facilitate bacterial spread by breaking down host tissue and counteracting neutrophil extracellular traps (NETs). Immune evasion is further supported by the production of enzymes that interfere with complement functions, like the cleavage of chemokines and the targeting of antibodies. Together, these virulence determinants allow GAS to cause a wide spectrum of diseases, ranging from uncomplicated pharyngitis and impetigo to invasive conditions like necrotising fasciitis and sepsis. This review provides a timely overview of the important GAS virulence factors and an update on the current vaccine landscape.

## 1. Introduction

Group A Streptococcus (GAS, *Streptococcus pyogenes*) is a Gram-positive exclusively human pathogen that is responsible for a wide range of diseases. These include skin and soft tissue infections, like pharyngitis, impetigo and erysipelas [1], and more invasive diseases such as necrotising fasciitis [2] and streptococcal toxic shock syndrome [3]. There has been a notable global increase in the incidences of severe invasive GAS infections since the late 1980s [4,5]. Around 15 years ago, scarlet fever, a disease associated with significant morbidity and mortality that had almost completely disappeared by the end of the twentieth century, re-emerged with outbreaks reported in China, Hong Kong, South Korea, Singapore, and the United Kingdom [6,7,8]. Untreated pharyngitis/tonsillitis can also lead to autoimmune sequelae acute rheumatic fever (ARF) and rheumatic heart disease (RHD) [9,10]. Another autoimmune complication, acute glomerulonephritis (AGN), is associated with impetigo [11].

It was estimated that a minimum of 18.1 million people suffer from severe GAS diseases, with an additional 1.78 million incident cases occurring each year. Annually, over 517,000 people die from severe GAS infections. In addition, over 111 million cases of streptococcal pyoderma and 616 million cases of GAS pharyngitis are reported each year [12,13]. Although invasive GAS infection rates dropped during the COVID-19 pandemic, a sharp increase in incidence and severity of invasive GAS infections was reported after the pandemic [1,14,15,16].

The greatest burden of GAS disease is due to RHD, with a prevalence of at least 15.6 million cases, 282,000 new cases, and 233,000 deaths each year [13].

GAS genes that encode M protein and T antigens display the highest levels of sequence diversity, giving rise to the two primary serological typing schemes historically used to define strains, as well as the subsequent genotyping system that is more commonly used nowadays. The N-terminal amino acid sequence of the surface-exposed M protein is epidemiologically classified into more than 100 M serotypes, which show differing patterns of regional and global distribution. However, serotyping has been mostly replaced by *emm*-genotyping, with over 200 *emm*-types [17]. T-typing is based on the trypsin-resistant heteropolymers (T antigens) that form the elongated pilus fibre [18]. Recently, a new genotyping scheme for pilus adhesin and pilus fibre (T antigen) genes was developed and combined with *emm* typing to provide a more accurate account of the global GAS strain population [19].

There is currently no licenced vaccine against GAS, but several vaccine candidates are in the development pipeline, and some have passed early clinical trials [20,21]. GAS is generally treatable with standard antibiotics, but there have been reports about increasing resistance to macrolides and tetracycline. High macrolide resistance is mainly due to ribosomal target modifications encoded by *erm* genes, ribosomal alterations, and active efflux pumps that regulate antibiotic entry due to *mefA/E* and *msrD* genes. Tetracycline resistance arises from *tet* gene expression, whereas combined tetracycline/macrolide resistance is usually linked with the insertion of *ermB* into the transposon carrying *tetM* [22]. GAS is generally still highly sensitive to beta-lactam antibiotics such as penicillin. However, two related GAS strains have been identified that showed reduced susceptibility to ampicillin, amoxicillin, and cefotaxime [23]. In a follow-up study, 137 geographically widespread GAS strains were found to carry mutations in the *pbp2x* gene, encoding penicillin-binding protein 2X (PBP2X), which has a lower affinity for beta-lactams [24].

GAS produces a large arsenal of secreted and cell surface-associated virulence factors that contribute to the pathogenesis of infection. These include adhesins, cytolysins, superantigens, spreading factors, and immune evasion factors. This review provides an overview of GAS virulence factors with a focus on novel insights and the vaccine landscape reported over recent years.

## 2. Secreted GAS Virulence Factors

**Figure 1:**
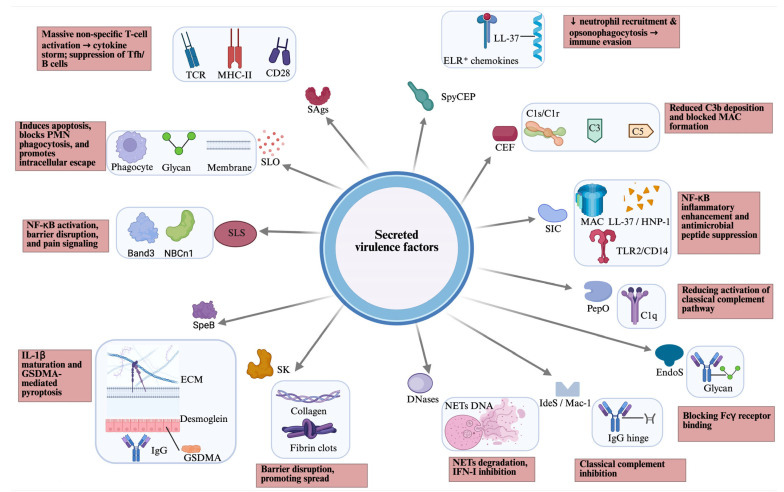
Overview of secreted virulence factors of GAS, including their host targets (blue box) and host effects (pink box). CEF, complement evasion factor; CD, cluster of differentiation; EndoS, endoglycosidase S; ECM, extracellular matrix; GAS, Group A Streptococcus; GSDMA, gasdermin A; HNP, human neutrophil peptide; IdeS/Mac-1, IgG-degrading enzyme of *S. pyogenes*; MHC, major histocompatibility complex; NETs, neutrophil extracellular traps; PepO, endopeptidase O; PMN, polymorphonuclear leukocytes; SAgs, superantigens; SLO, streptolysin O; SLS, streptolysin S; SpeB, streptococcal cysteine protease B; SK, streptokinase; SIC, streptococcal inhibitor of complement; SpyCEP, *Streptococcus pyogenes* cell envelope protease; TCR, T cell receptor; TLR, toll-like receptor. Created in BioRender. Fan, S. (2026). https://BioRender.com/mmq4i6x.

GAS secretes a large arsenal of virulence to evade immunity, degrade tissue, and spread, including lysing host cells, disabling immune signals (chemokines and complement), breaking down neutrophil extracellular traps (NETs), and causing systemic effects like toxic shock (Figure 1).

### 2.1. Superantigens (SAgs)

Superantigens (SAgs) are a family of structurally related T cell mitogens produced by GAS and a few other Gram-positive cocci. A hallmark of SAgs is their ability to simultaneously bind to major histocompatibility complex (MHC) class II on antigens presenting cells and the T cell receptor (TcR) on T cells. In contrast to conventional peptide antigens, superantigens bind to MHC class II outside the peptide-binding groove, and sequentially bind the TcR via the variable region of the TcR b-chain, stimulating up to 25% of an individual’s T cell population. The oligoclonal stimulation of T cells and antigen-presenting cells (APCs) by SAgs results in a massive release of pro-inflammatory cytokines, such as tumour necrosis factor (TNF), interleukin 1-beta (IL-1b), and T cell mediators, such as IL-2, which can lead to fever and shock [25]. CD28, the general co-stimulatory receptor, which is constitutively expressed on T cells and interacts with B7 molecules (CD80 and CD86), has been identified as an additional and essential receptor for cytokine production [26]. It was also shown that SAgs that bind to the MHC class II b-chain, such as SMEZ, also bind directly to CD28 and B7, enhancing B7/CD28 costimulatory axis formation, which is crucial for T cell activation [27]. SAg activation has also been shown to engage mucosal-associated invariant T (MAIT) cells, which respond by releasing high levels of pro-inflammatory cytokines [28]. Streptococcal SAgs have been implicated in a range of human diseases, most notably, toxic shock syndrome and scarlet fever [29]. Currently, there are 13 known superantigens produced by GAS, which include the streptococcal pyrogenic exotoxins (Spe) A, C, G-M, R, and Q, streptococcal superantigen (SSA), and streptococcal mitogenic exotoxin Z (SMEZ) [29]. Three of them (SpeA, SpeC, and SSA) have been associated with increased fitness and virulence of contemporary GAS strains causing scarlet fever and invasive disease [6,30]. Those strains harbour a prophage that, in addition to *sag* genes, also carries genes expressing the secreted DNase Spd1 and the cytolysin streptolysin O (SLO). Spd1 was found to act synergistically with SpeC to facilitate nasopharyngeal colonisation, whereas SLO induced glutathione efflux, which promotes SSA release and activity [30]. A recent study has shown that a new variant of the GAS serotype M1 (designated ‘M1UK’), which has been linked with seasonal scarlet fever surges in the U.K., exhibited enhanced expression of SpeA. This was due to a single SNP in the 5′ transcriptional leader sequence of the transfer-messenger RNA gene *ssrA* that drives enhanced SpeA expression as a result of *ssrA* terminator read-through [31].

SpeA was also shown to play an important role in acute nasopharyngeal infection using transgenic mice that express human MHC class II molecules as a SAg-sensitive infection model, where GAS infection was strongly enhanced in mice that express HLA-DQ8 or HLA-DR4 [32]. Furthermore, SAg-dependent, Vβ8-specific T cell activation in the cervical lymph nodes was observed during *GAS* infection and these Vβ8-specific T cells were required for efficient nasopharyngeal infection, as mice vaccinated with SpeA rendered Vβ8-specific T cells poorly responsive, resulting in reduced infection [33].

In tonsillar tissue, SpeA exposure triggers B cell apoptosis and markedly reduces Ig production, thereby weakening the humoral immune response. In SpeA-stimulated cultures, T follicular helper cells downregulated C-X-C chemokine receptor 5 while upregulating OX40, an inducible T cell co-stimulator. This altered Tfh phenotype correlated with impaired chemotaxis toward CXCL13. These findings suggest that SAg-driven dysregulation of tonsillar immunity redirects Tfh cells toward a proliferative state, leading to B cell loss and diminished antibody production, changes that likely confer a survival advantage to superantigen-producing bacteria [34].

It has been known for about two decades that streptococcal SAgs exhibit varying susceptibility to degradation by the cysteine protease SpeB. While SMEZ is most sensitive to degradation, other SAgs, like SpeA and SpeG, are more resistant and SpeJ is completely unaffected [35]. A more recent study demonstrated that SpeA is not only protease-resistant but works synergistically with SpeB to induce inflammation [36].

Despite their strong association with toxic shock syndrome, GAS SAgs do not appear to be the primary drivers of invasion but seem to function predominantly as amplifiers of pathology once invasive infection has been established. SAgs are neither necessary nor sufficient for tissue invasion and are absent from many severe invasive isolates. Their contribution appears temporally and contextually constrained, requiring high bacterial burden and systemic exposure, and might be best understood as potentiating host immune dysregulation rather than initiating disease.

### 2.2. Cytolysins and NAD Glycohydrolase

#### 2.2.1. Streptolysin O (SLO)

SLO belongs to the family of cholesterol-dependent pore-forming toxins which are found predominantly in Gram-positive pathogens. Pore formation is due to the self-assembly of monomers bound individually to cholesterol-rich membranes, although alternative receptors also contribute. SLO binds to a broad range of glycans and the highest affinity interactions were detected for the ABO blood group antigens, with the highest affinity observed for the group B type IV pentasaccharide [37]. Receptor binding is followed by dimerisation and oligomerisation, inducing rapid, dose-dependent apoptosis in many human cells, in particular macrophages and neutrophils, and protecting the bacteria from phagocytic killing [38]. SLO promotes bacterial escape from the GAS-containing vacuole (GCV) into the macrophage cytosol and cytosolic growth, despite autophagy receptor recruitment [39]. In neutrophils, SLO has the capacity to suppress oxidative burst and other key neutrophil functions, including degranulation, directed migration, and formation of NETs, together promoting neutrophil resistance [40]. SLO functions synergistically with NAD glycohydrolase (NADase), a co-expressed enzyme that depletes the cellular energy stores of host cells. The two toxins form stable complexes in solution, enhancing SLO-mediated cytotoxicity and contributing to GAS virulence [41]. Moreover, SLO and NADase disrupt host cell physiology by inducing Golgi fragmentation and inhibiting anterograde transport, thereby increasing bacterial translocation across epithelial barriers [42].

Cyclic-di-AMP produced by GAS can pass through streptolysin O pores into macrophages, where it activates a stimulator of interferon genes (STING) and triggers a type I IFN response. However, this response is suppressed by invasive strains expressing enzymatically active NADase. Patient analyses from necrotising GAS soft tissue infections show that a STING variant with reduced cyclic-di-AMP binding, combined with high bacterial NADase activity, creates a “perfect storm” associated with poor outcomes. In contrast, efficient and uninhibited STING-mediated type I interferon (IFN) responses are linked to protection against harmful inflammation [43].

The central role of SLO in GAS pathogenicity is further underscored by hypervirulent strains that produce elevated levels of the toxin, resulting in enhanced hemolysis, reduced dendritic cell (DC) viability, and increased TNF and MCP-1 production by DCs [44].

#### 2.2.2. Streptolysin S (SLS)

SLS is a 2.7 kDa ribosomally synthesised, post-translationally modified peptide with a broad cytolytic spectrum, which includes erythrocytes, leukocytes, platelets, and even sub-cellular organelles. SLS is responsible for the typical β-hemolytic phenotype seen around colonies of GAS, when cultured on blood agar [45]. SLS induces osmotic lysis of red blood cells by disrupting the chloride–bicarbonate exchanger band 3, leading to rapid Cl^−^ influx and subsequent cell rupture [46]. More recently, SLS has also been shown to target the electroneutral sodium–bicarbonate cotransporter NBCn1 in keratinocytes, triggering NF-κB activation and cytotoxicity during GAS infection [47]. The importance of SLS in colonisation and disease has been highlighted by studies using SLS-deficient strains, which showed a 100-fold reduction in bacterial recovery from the nasopharynx and a 10-fold reduction in bacterial burden in skin. Neutrophil depletion partially restored bacterial load in the skin but not in the nasopharynx, suggesting tissue-specific roles. In mice infected intranasally with wild-type GAS, SLS caused relocalisation and disruption of the tight junction protein ZO-1 at sites of infection, a change absent in infections with SLS-deficient strains, underscoring its role in epithelial barrier damage [48]. Beyond epithelial disruption, SLS induces macrophage death by preventing glycogen synthase kinase-3β (GSK-3β) degradation and exacerbating mitochondrial damage [49]. SLS also contributes to immune evasion by perforating the phagolysosomal membrane to allow leakage of protons and proteins like cathepsin B [50]. It also activates nociceptor neurons, driving pain responses during infection, particularly in necrotising fasciitis. Activated nociceptors release calcitonin gene-related peptide (CGRP) into infected tissues, which suppresses neutrophil recruitment and impairs opsonophagocytic killing of GAS [51].

### 2.3. Streptococcal Pyrogenic Exotoxin B (SpeB)

Streptococcal pyrogenic exotoxin B (SpeB) was originally mis-classified as a superantigen due to contamination of the purified sample and has been re-classified as streptococcal cysteine protease (or streptopain). SpeB has a very broad substrate spectrum and degrades many proteins secreted by GAS, including other virulence factors, like SMEZ [35] (see above). In addition, SpeB has been shown to degrade the extracellular matrix, cytokines, chemokines, complement components, immunoglobulins, and serum protease inhibitors (reviewed in [52,53]). SpeB cleaves the extracellular domains of desmoglein-1 and -3, molecules critical for tissue integrity and cell–cell communication. In an epicutaneous infection model, lesions caused by an *speB* deletion mutant were significantly smaller than those produced by the wild-type strain, indicating that SpeB-mediated desmosome degradation plays a pathogenic role in the development of GAS skin infections [54]. Furthermore, SpeB modulates host immune responses by targeting multiple factors. Notably, it can directly activate IL-1β independently of canonical inflammasome pathways, thereby amplifying immune responses that limit GAS invasion [55]. SpeB cleaves multiple immunoglobulin classes (IgA, IgM, IgD, and IgE) and selectively digests antigen-bound IgG on bacterial surfaces through its Fab domain, while sparing IgG that is bound non-immunologically via Fc-binding proteins. By separating the Fc and Fab fragments of IgG, SpeB reduces antibody-mediated opsonophagocytosis of GAS in vitro [56]. Nevertheless, the in vivo significance of this antibody cleavage remains debated [57].

More recently, SpeB has been shown to trigger keratinocyte pyroptosis by cleaving Gasdermin A (GSDMA), a pore-forming member of the gasdermin family that acts as a sensor, substrate, and effector of pyroptosis. This cleavage releases an active N-terminal fragment that initiates pyroptosis. Caspase-independent cleavage of GSDMA by SpeB is highly selective and requires SpeB to enter the cytosol of the infected cell. In mice, genetic deficiency of *Gsdma1* impaired immune responses to GAS, leading to uncontrolled bacterial dissemination and death. Thus, GSDMA functions simultaneously as a sensor and substrate of SpeB, as well as an effector that induces pyroptosis, providing a streamlined, one-molecule mechanism for host recognition and control of a highly virulent pathogen [58,59].

### 2.4. Streptokinase (SK)

Streptokinase (SK) is a secreted protein that activates host plasminogen, converting it into plasmin. This fibrinolytic activity enables GAS to degrade fibrin clots and extracellular matrix components, facilitating bacterial spread through tissues and evasion of host immune defences. In contrast to other plasminogen activators, SK lacks intrinsic enzymatic activity. By hijacking the host’s own proteolytic system, SK enhances invasiveness and contributes to severe diseases such as necrotising fasciitis and streptococcal toxic shock syndrome. Its potent activity has also been harnessed clinically as a thrombolytic agent for dissolving blood clots in cardiovascular medicine, though its bacterial origin limits its widespread use today (reviewed in [60]). GAS recruits plasminogen and plasmin to its surface through several cell wall-associated proteins, including M proteins, glyceraldehyde-3-phosphate dehydrogenase, and streptococcal enolase. Together with streptokinase, these factors generate both soluble and surface-bound plasmin activity, enhancing the pathogen’s invasive potential. A more recent study has shown that SK acts synergistically with plasminogen-binding M protein (PAM) to enhance plasminogen activation, leading to fibrin clot dissolution and keratinocyte wound layer retraction, thereby facilitating bacterial dissemination [61]. Until recently, the mechanism by which GAS embedded within fibrin clots accessed plasminogen for dissolution was unclear. Live imaging studies by Vu and colleagues showed that bacteria entrapped in clots can persist in a dormant state until plasminogen becomes available to trigger fibrinolysis. Using a 3D endothelial microfluidic model, they further demonstrated that GAS can effectively induce fibrinolysis in an endothelial setting, enabling its dissemination into the bloodstream [62]. During the early stages of GAS infection, SK aids bacterial evasion of innate immunity by promoting plasmin-mediated degradation of host defence molecules, including the antimicrobial peptide LL-37, complement component C3b, and histones [63,64,65]. In addition, SK can trigger contact system activation at the bacterial surface, leading to cleavage of high-molecular-weight kininogen and release of bradykinin, thereby amplifying inflammation during infection [66].

### 2.5. Secreted DNases

A total of six prophage-encoded secreted DNase genes (*sda1*, *sda2*, *spd1*, *spd3*, *spd4*, and *sdn*) and one chromosome-encoded secreted DNase (*spdB*) have been identified in GAS. Each GAS strain secretes at least one extracellular DNase and most strains produce several of these enzymes. An isogenic triple mutant of a contemporary M1 strain was less virulent compared to the Wt strain in two mouse invasive disease models [67]. The important contribution of DNases to GAS disease progression was also demonstrated in a study that used *Δspd* and *ΔsdaD2* deletion mutants to show that infected mice survived better than those infected with WT GAS and that degradation of the DNA backbone structure of NETs plays an important role [68]. Furthermore, DNA degradation by Sda1 destroyed CpG-rich DNA, a ligand for toll-like receptor 9 (TLR9). This prevented IFN-α and TNF secretion from murine macrophages, indicating that Sda1 suppresses TLR9-mediated immune responses and macrophage bactericidal activity [69]. Sda1 impairs plasmacytoid dendritic cell recruitment to the infected tissue by reducing IFN type-1 levels at the site of infection. It was also observed that Sda1 interferes with stabilisation of the DNA by the host molecule High-Mobility Group Box 1 (HMGB1) protein, which may account for decreased IFN type-1 levels at the site of infection [70].

A recent epidemic serotype M3 strain that has acquired a prophage expressing Spd1 and the superantigen SpeC showed enhanced nasopharyngeal infection and shedding and this was, in part, mediated by the gain of the *spd1* gene [71]. Spd1 is co-expressed with the phage-encoded superantigen SpeC and the carriage of a prophage encoding superantigens SSA and SpeC, and Spd1 has been associated with scarlet fever outbreak strains in Hong Kong, Mainland China, and the United Kingdom [6,7].

### 2.6. Complement Evasion Factors

The human complement system is a network of plasma proteins and cell surface receptors that form a key part of the innate immune system. When activated, it enhances the ability of antibodies and phagocytes to clear microbes and damaged cells by promoting opsonisation, inflammation, and direct lysis of pathogens. Complement can be triggered through three main pathways (classical, lectin, and alternative), all converging on the activation of C3 and formation of the membrane attack complex (MAC) (reviewed in [72]).

GAS secretes several immune evasion factors that interfere with complement function, enabling the bacteria to escape clearance.

#### 2.6.1. Immunoglobulin-Degrading Enzyme of *S. pyogenes* (IdeS)

The immunoglobulin-degrading enzyme of *S. pyogenes* (IdeS, also known as macrophage antigen-1, Mac-1) is a cysteine endopeptidase that specifically cleaves human and rabbit IgG1–4 within the heavy chain, producing circulating F(ab’)_2_ fragments [73]. Although these fragments can still bind to the bacterial surface, they are unable to activate complement [74]. An allelic variant, Mac-2, was initially reported to have weak endopeptidase activity to IgG, instead binding to Fcγ receptors, thereby competitively inhibiting IgG binding [75]. Subsequent work showed this variant was restricted to serotype M28 strains, and that Mac-2 from other serotypes retained IgG endopeptidase activity as well as Fcγ receptor interaction [76]. The recently solved crystal structure of IdeS/Mac-1 revealed binding across both Fc chains, spanning the entire hinge region, with largely conserved Fc-interacting residues between Mac-1 and Mac-2, consistent with their overlapping activities [77]. Despite this mechanistic detail, the contribution of IdeS to immune evasion in vivo remains uncertain, as deletion of *mac/ideS* in the hyperinvasive M1/T1 strain had no measurable effect on phagocytic uptake, oxidative burst, resistance to killing, or virulence in mouse models [78]. However, recent work showed that IdeS forms proteolytically active complexes with the cell wall-anchored nuclease SpnA on the GAS surface and that IgG binding to GAS surface antigens was efficiently cleaved by surface-bound IdeS, suggesting an important role in pathogenesis by inhibiting the classical pathway of complement [79].

#### 2.6.2. Endo-β-N-acetylglucosaminidase of *S. pyogenes* (EndoS)

The endo-β-N-acetylglucosaminidase of *S. pyogenes* (EndoS) is an endoglycosidase that specifically targets the IgG heavy chain by cleaving the chitobiose core of its N-linked glycan. Because this glycan is essential for Fcγ receptor binding, EndoS modification interferes with IgG-mediated complement activation. Pre-incubation of opsonising IgG with recombinant EndoS enhanced GAS survival in ex vivo human blood assays, supporting a role in immune evasion [56]. However, its in vivo relevance remains elusive. Deletion of *endoS* in an M1/T1 strain had no effect on survival in immune cell-killing assays or in systemic mouse infection, while heterologous expression in an M49 background increased virulence, suggesting strain-specific regulation of EndoS expression [80]. Structural studies have provided detailed mechanistic insight. A co-crystal structure revealed that the EndoS glycosidase domain traps the IgG1 Fc glycan in a “flipped-out” conformation, with additional peptide recognition through a carbohydrate-binding module [77]. More recently, cryoEM analysis demonstrated that EndoS exhibits strict protein-specificity, requiring protein–protein interactions through a non-enzymatic domain to anchor to IgG. Hydrolysis occurs sequentially on the two Fc glycans of the IgG homodimer through a catch-and-release mechanism [81].

Like IdeS, EndoS shows strong biochemical activity in vitro but only modest or context-dependent phenotypes in animal models. This discrepancy likely reflects redundancy and buffering within GAS immune evasion networks rather than biological irrelevance; therefore, these enzymes might be better viewed as immune-modulating factors that fine-tune host–pathogen interactions rather than classical virulence factors.

#### 2.6.3. Endopeptidase O

GAS expresses an endopeptidase O (PepO) orthologue sharing 68% amino acid identity with the pneumococcal PepO protein, which binds C1q through electrostatic interactions and, under low pH conditions, interferes with its binding to IgG and short pentraxins such as C-reactive protein (Honda-Ogawa et al., 2017 [82]). C1q is a component of the initiation complex of the classical complement pathway. Deletion of *pepO* reduced resistance to phagocytic killing and attenuated virulence in a mouse model of invasive infection (Honda-Ogawa et al., 2017). In addition, PepO was shown to influence growth phase-dependent expression of SpeB in an M1/T1 strain, with *pepO* mutants exhibiting increased susceptibility to neutrophil-mediated killing and reduced virulence in mice (Brouwer et al., 2018 [83]).

#### 2.6.4. Streptococcal Inhibitor of Complement (SIC)

The streptococcal inhibitor of complement (SIC) is uniquely expressed by the highly virulent M1/T1 strain of GAS. Functionally, SIC binds to the soluble C5b-9 complex also known as the membrane attack complex (MAC), thereby blocking its insertion into the bacterial membrane [84]. In Gram-negative bacteria, MAC disrupts membranes by forming lytic pores. In Gram-positive bacteria, however, MAC activity is less clear, as the thick peptidoglycan cell wall is thought to hinder access to the membrane. Interestingly, in GAS C5b-9 deposition, is not random but occurs at distinct sites near the division septum, unlike the diffuse pattern of C3b deposition. This suggests that during certain growth phases, structural remodelling of the cell wall may render the bacteria vulnerable to MAC insertion [85]. Beyond complement inhibition, SIC also blocks human neutrophil α-defensin (HNP-1) and LL-37, two major antibacterial peptides involved in bacterial clearance [86]. More recently, SIC was shown to interact with TLR2 and CD14 on monocytes, activating the NF-κB and p38 MAPK pathways and driving the release of pro-inflammatory cytokines such as TNF and IFNγ. In human plasma, SIC binds to clusterin and histidine-rich glycoprotein, and both proteins, whether in purified form or within whole plasma, enhance SIC-mediated monocyte activation [87]. Notably, while native SIC secreted from the bacteria generates non-protective antibodies, vaccination with full-length recombinant SIC permits the development of protective antibodies [88]. However, thus far, SIC has not been further evaluated as a potential vaccine candidate.

#### 2.6.5. Complement Evasion Factor (CEF)

The recently characterised complement evasion factor (CEF), encoded by *spy0136*, is a secreted protein that binds several key complement components, including C1r, C1s, C3, and C5, with some interactions dependent on N-linked glycans. Through these interactions, CEF interferes with all three complement activation pathways, reducing C3b deposition, inhibiting MAC formation, and preventing complement-mediated hemolysis. Deletion of *cef* in an M1 strain reduced bacterial survival in human whole blood, demonstrating its role in infection. CEF may disrupt C1 complex formation or inhibit the enzymatic activity of C1r and/or C1s, thereby preventing C4 cleavage and formation of the classical pathway C3 convertase, C4bC2a. However, the precise molecular mechanisms of complement inhibition and the specific domains responsible for binding remain elusive [89].

## 3. Cell Surface-Associated Virulence Factors

**Figure 2:**
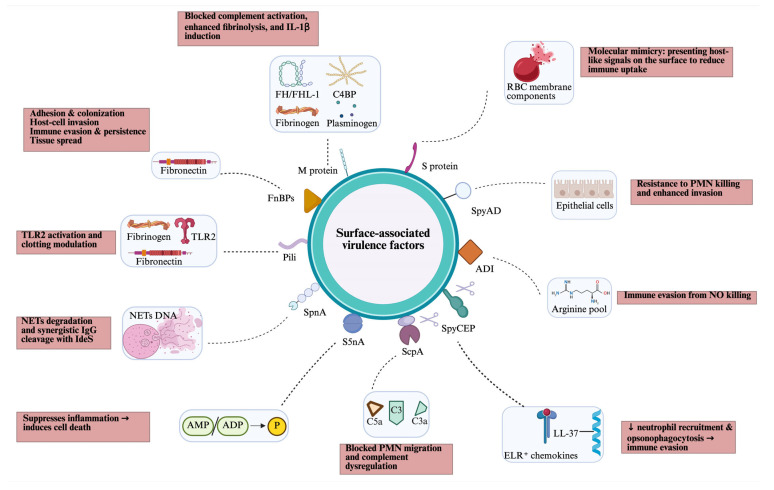
Overview of cell surface-associated virulence factors of GAS, including their targets (blue box) and host effects (pink box). ADI, arginine deiminase; AMP/ADP, adenosine monophosphate/adenosine diphosphate; C4BP; C4-binding protein; FH/FHL, factor H/factor H-like; FnBPs, fibronectin-binding proteins; NETs, neutrophil extracellular traps; PMN, polymorphonuclear leukocytes; RBC, red blood cell; SpnA, *Streptococcus pyogenes* nuclease A; S5nA, streptococcal 5-nuclease A; ScpA, C5a peptidase; SpyCEP, *Streptococcus pyogenes* cell envelope protease; SpyAD, *Streptococcus pyogenes* adhesion and division protein; TLR, toll-like receptor. Created in BioRender. Fan, S. (2026). https://BioRender.com/mmq4i6x.

GAS produces several surface-associated virulence factors, most of which are anchored to the cell wall by the transpeptidase sortase A (SrtA), which recognises a specific anchor motif (LPXTG) at the start of a C-terminal hydrophobic cell wall-anchor domain. Some cell wall-anchored proteins, such as C5a peptidase and SpyCEP, are also partly released as soluble forms after SpeB protease cleavage (Figure 2).

### 3.1. M Protein

M protein is a α-helical coiled-coil protein that extends outward from the bacterial cell wall and projects beyond the thick capsule, where it can directly interact with host components [90]. M protein is encoded by the *emm* gene, which is highly polymorphic, giving rise to more than 250 distinct *emm* types. These variants are used in molecular epidemiology to classify GAS strains [17].

An important role of M protein is to protect GAS against innate immune defences, particularly phagocytosis, which is achieved through multiple mechanisms. For example, M protein binds host regulators of complement, such as factor H (FH), factor H-like protein 1 (FHL-1), and C4b-binding protein (C4BP), thereby interfering with complement activation and reducing opsonisation, although binding often differs between *emm* types. Factor H regulates complement activation by acting as a cofactor for factor I-dependent cleavage of C3b and by disrupting the alternative pathway C3 convertase, whereas C4BP binds to C4b and is involved in the regulation of the classical pathway by accelerating the decay of the C3 convertase and also by acting as a cofactor in the factor I-mediated proteolytic inactivation of C4b (reviewed in [91]). Although the interaction between factor H and M proteins is well established, its relevance under physiological conditions remains controversial. A more recent study using transgenic mice failed to establish a clear role for M protein-bound FH during acute infection. Moreover, phagocytosis assays indicated that the ability to bind FH is neither sufficient nor necessary for GAS to resist killing in whole human blood [92]. M protein can also bind fibrinogen and plasminogen. Fibrinogen binding by M protein reduces the amount of classical pathway C3 convertase, and, hence, C3b deposition on the bacterial surface [93], whereas bound plasminogen can be activated by exogenous and endogenous streptokinase, thereby providing the bacteria with a surface-associated protease that is able to destroy deposited complement proteins [94]. Furthermore, plasminogen/plasmin binding promoted integrin-mediated internalisation of an M49 strain into keratinocytes, with α(1)β(1)- and α(5)β(1)-integrins acting as the major keratinocyte receptors [95]. More recently, it was shown that a soluble form of the M1 protein triggers cell death in macrophages by working as a second signal for caspase-1-dependent NLRP3 inflammasome activation. This resulted in the release of IL-1β and macrophage pyroptosis [96].

M protein also contributes to type-specific adherence and colonisation via adhesive interactions with cell-surface glycans and extracellular matrix proteins like fibronectin, facilitating bacterial persistence in the upper respiratory tract and skin. The M1 protein has high affinity for several terminal galactose blood group antigen structures and an M1 strain showed higher adherence to oral epithelial cells that expressed H antigen structures compared to cells expressing A, B, or AB antigen structures. The role of fibronectin binding in bacterial host cell adhesion has been well documented (reviewed in [97]). It has recently been demonstrated that human antibodies increase fibronectin binding to M proteins, which leads to a reduction in antibody-mediated phagocytosis, indicating another immune evasion mechanism [98].

### 3.2. Fibronectin-Binding Proteins

At least 11 fibronectin-binding proteins (FNBPs) have been identified in GAS, and they fall into two major categories. The first group, including Protein F1 (PrtF1/SfbI), Protein F2 (PrtF2/PFBP), FbaA, FbaB, SfbII/serum-opacity factor (SOF), SfbX, and Fbp54, contains characteristic fibronectin-binding repeat domains. The second group, which includes M1 protein, GAPDH/Plr, Shr, and Scl1, lacks these repeat motifs. Most GAS strains carry Fn-binding proteins with repeat-containing domains and a C-terminal LPXTG cell wall-anchoring motif; the specific repertoire of Fn-binding proteins present in a strain closely correlates with its M serotype.

By physically linking the bacterium to host fibronectin, a ubiquitous extracellular matrix glycoprotein present on epithelial surfaces, in plasma, and in the basement membrane, FnBPs convert passive contact into active colonisation, invasion, and dissemination. Their contribution to virulence can be understood across four interlocking functions: adhesion and colonisation, host cell invasion, immune evasion and persistence, and facilitation of tissue spread. For a more detailed overview, we refer to the review article by Yamaguchi and colleagues [99,100,101].

### 3.3. Pili

Pili are long, cell wall-anchored, hair-like structures protruding from the GAS cell surface. The pilus structure consists of two or three structural proteins assembled covalently by a specialised sortase enzyme, including the tip adhesin, a homo-polymerised fibre protein, and (with some exceptions) a cell wall-anchor protein. The fibre protein is also known as the T antigen, which is often used in strain typing. Pili play important roles in host cell adhesion, and some pilus types also contribute to biofilm formation (reviewed in [102]). Some pilus types also contribute to immune evasion. The tip protein of the M2/T2 pilus lacks adhesive properties and, instead, the fibre pilin is important for host cell adhesion, binding several host factors, including fibronectin and fibrinogen. This pilus contributed to a delay in blood clotting, increased intracellular survival of the bacteria in macrophages, higher bacterial survival rates in human whole blood, and greater virulence in a *Galleria mellonella* (Great wax moth) infection model [103]. Similarly, the fibre protein of the M4/T4 pilus binds the human acute-phase protein haptoglobin, which reduces the susceptibility of M4 GAS to antimicrobial peptides released from activated neutrophils and platelets, resulting in increased survival in human whole blood and increased virulence in murine models of invasive infection [104].

More recently, we have demonstrated that GAS pili and individual pilus proteins induce a potent release of TNF and IL-8, and that the tip and fibre proteins of the M1/T1 strain are toll-like receptor-2 (TLR-2) agonists [105]. There are differences between pilus types, with M6/T6 pili inducing stronger cytokine responses than other pili. Notably, there was a negative correlation between the observed immune responses and disease severity in the GAS strains associated with individual pilus types, suggesting that pili may not be a major contributor to inflammatory symptoms but instead are more likely to contribute to bacterial clearance [106].

### 3.4. Streptococcus pyogenes Nuclease A (SpnA) and Streptococcal 5′-Nucleotidase A (S5nA)

*Streptococcus pyogenes* nuclease A (SpnA) is the only known surface-attached nuclease in GAS. It promoted survival in whole human blood and in a neutrophil killing assay, which was facilitated by the destruction of NETs. Furthermore, a *spnA* deletion mutant was less virulent than the parental strain in a mouse infection model [107,108]. SpnA consists of two major domains, a C-terminal nuclease domain and an N-terminal domain, containing three oligosaccharide/nucleotide-binding (OB) fold motifs, which are believed to play a role in substrate binding. Notably, abolishing the enzymatic activity of SpnA only partially reduced virulence, suggesting that SpnA has an additional virulence function, which might be located on the N-terminal domain [109]. As described earlier, SpnA forms a complex with the secreted complement evasion factor IdeS, resulting in cleavage of surface-bound IgG, which inhibits the classical pathway of complement [79].

Streptococcal 5′-nucleotidase A (S5nA) is a cell wall-anchored 5′-nucleotidase that hydrolyses AMP and ADP to generate adenosine, which suppresses host inflammatory responses by inhibiting cytokine production and modulating leukocyte chemotaxis. S5nA also hydrolyses dADP to produce deoxyadenosine, which is toxic for macrophages. As dADP is a nuclease cleavage product from DNA, S5nA might work in synergy with SpnA. The role of S5nA in virulence was further demonstrated by increased survival of non-pathogenic *L. lactis* bacteria in the presence of recombinant S5nA during a whole blood killing assay [110].

### 3.5. Neutrophil Chemotaxis-Interfering Enzymes

#### 3.5.1. C5a Peptidase (ScpA)

GAS produces C5a peptidase (ScpA), a subtilisin-like serine protease that is anchored to the bacterial cell wall but can also be released into the environment through the action of the broad-spectrum protease SpeB [111]. ScpA specifically cleaves the C-terminus of C5a, completely abolishing its chemotactic activity to attract phagocytes [112]. The physiological role of ScpA was demonstrated in a mouse air sac model, where an *scpA* deletion mutant was cleared more efficiently than the wild-type strain and induced greater infiltration of polymorphonuclear leukocytes (PMNLs) [113]. The crystal structure of active ScpA has been solved, and modelling of the ScpA–C5a complex suggests an interaction that closely mimics native C5a receptor–ligand binding [114]. Asp130, His193, and Ser512 have been identified as catalytic site residues and alanine conversion has resulted in a >1000-fold reduction in enzymatic activity [115]. Those toxoids are currently used in GAS vaccine formulations (Section 4). More recently, Sriskandan and colleagues showed that ScpA can also cleave C3 and C3a. This activity produces aberrant C3a and C3b fragments with impaired function and reduces neutrophil activation [116].

#### 3.5.2. *Streptococcus pyogenes* Cell Envelope Protease (SpyCEP)

GAS expresses the subtilisin-like protease SpyCEP, which is anchored to the cell wall by sortase but is also released from the surface in the later growth phase [117].

SpyCEP cleaves CXC chemokines containing an N-terminal Glu-Leu-Arg motif (ELR^+^ chemokines), including the neutrophil chemoattractant CXCL8 (IL-8), at a distal C-terminal site within the chemokine. This contributes to the suppression of neutrophil migration into the site of infection and, consequently, the evasion of opsonophagocytosis [117,118]. Mice infected with a *spyCEP* deletion mutant generated strongly increased amounts of the chemokines KC (murine functional homologue of IL-8), LIX (CXCL5 in humans), and MIP-2 (Macrophage Inflammatory Protein 2, CXCL2) [117]. Recently, the importance of the ELR motif was further demonstrated by swapping the N-terminal regions of the ELR^+^ chemokine IL-8 and the ELR^−^ chemokine CXCL4, resulting in loss-of-function (IL-8) and gain-of-function (CXCL4) substrates. Furthermore, alanine substitution of the IL-8 ELR motif impaired substrate binding and enzyme kinetics [119]. SpyCEP is also active against the human antimicrobial peptide LL-37, cleaving it into two fragments, which results in the inhibition of neutrophil chemotaxis, a shortened neutrophil lifespan, and blockage of purinergic P2X7 and Epidermal Growth Factor (EGF) receptor activation [120].

The catalytic triad of SpyCEP was identified as Asp151, His279, and Ser617. The double mutant D151A/S617A was shown to be catalytically inactive [121], yet it was able to elicit functional anti-SpyCEP antibodies and provide protection in a mouse intranasal infection model [122], demonstrating potential use in GAS vaccine formulations (Section 4).

### 3.6. Arginine Deiminase (ADI)

Arginine deiminase (ADI) hydrolyses arginine into citrulline and ammonia as part of the arginine deiminase pathway. Although a cytoplasmic enzyme, the protein has also been located on the cell surface [123]. ADI might contribute to immune evasion by depleting arginine, which host immune cells need for nitric oxide (NO) production, thus dampening NO-mediated bacterial killing [124].

A GAS serotype M49 *arcA* knockout mutant lacking arginine catabolism resulted in 5-fold higher arginine levels in human PMBC supernatants compared to the parent strain and approximately two-fold higher mitochondrial dehydrogenase activity and increased IL-2 production. Furthermore, ADI lowered the proportion of central memory (CM) T cells while increasing terminally differentiated effector memory (TEMRA) populations within both CD4^+^ and CD8^+^ subsets. ADI activity also inhibited proliferation across all CD8^+^ T cell subsets, as well as CM, EM, and TEMRA CD4^+^ T cells. A notable effect of ADI was the suppression of autophagy in CD8^+^ CM and effector memory (EM) cells, and in CD4^+^ CM, EM, and TEMRA subsets. This suggests that ADI can dampen adaptive immunity and weaken immunological memory [125].

### 3.7. Streptococcus pyogenes Adhesion and Division Protein (SpyAD)

The membrane-bound *Streptococcus pyogenes* adhesion and division protein (SpyAD, previously Spy0269) plays a role in both cell division and host cell adhesion. Deletion of the *spyAD* gene in a GAS M1 strain resulted in very long bacterial chains, indicating impaired cell division. Recombinant SpyAD bound to mammalian epithelial cells and a *Lactococcus lactis* gain-of-function mutant was able to bind to mouse nasal mucosa [126]. Recently, it was demonstrated that SpyAD is critical for GAS invasion of human pharyngeal and vaginal epithelial cells, facilitating bacterial persistence. Furthermore, SpyAD enhanced resistance to neutrophil-mediated killing and increased bacterial survival in a mouse systemic infection model [127].

### 3.8. S Protein

The S protein is a recently identified highly conserved GAS virulence factor found to be both cell membrane-bound and secreted. The S protein promotes immune evasion through molecular mimicry by facilitating the coating of the GAS surface with lysed red blood cells. Deletion of the *ess* gene resulted in reduced survival in human blood and increased immune response and immunological memory [128].

### 3.9. Group A Carbohydrate (GAC)

The cell wall-anchored Group A Carbohydrate (GAC) is universally expressed by all GAS strains and therefore has been used in GAS serological classification as well as clinical diagnosis. GAC is a polymer mainly formed by a polyrhamnose backbone with N-acetylglucosamine (GlcNAc) sidechains and plays an important role in stabilising the cell wall. Although the exact virulence mechanisms remain elusive, an isogenic mutant of the glycosyltransferase *gacI*, which encodes for an enzyme that adds the GlcNAc sidechain, was shown to be attenuated for virulence in a murine systemic infection model and a rabbit pulmonary infection model. Lack of GlcNAc was associated with increased sensitivity to neutrophil killing, platelet-derived antimicrobials in serum, and the cathelicidin antimicrobial peptide LL-37 [129]. The immunogenicity, surface accessibility, and strong conservation between GAS strains make GAC a promising vaccine candidate (see Section 4). A drawback is that data reported more than five decades ago found higher levels and greater persistence of antibodies to GAC among ARF patients with carditis compared to matched controls, which was believed to be due to molecular mimicry with GlcNAc [130]. Consequently, GAC derivatives were developed consisting of the polyrhamnose backbone without the GlcNAc sidechain for the potential use in vaccines. However, more recently, it was suggested that while some ARF-associated autoantibodies react with GlcNAc sidechains of GAC and cross-react with tissues, these appear to be a consequence, not the cause, of autoimmunity [131].

## 4. Vaccine Development

Safety concerns have long represented a significant gap in the development of GAS vaccines. As early as 1969, a report described that 3 out of 21 volunteers developed ARF following immunisation with a partially purified M3 protein preparation, raising serious safety concerns about GAS vaccines and the theoretical risk of autoimmunity [132]. This historical incident significantly influenced subsequent GAS vaccine development, prompting a stronger focus on the evaluation of cross-reactive epitopes and the underlying immunological mechanisms. Consequently, it had a profound impact on the development of GAS vaccine research.

**Figure 3:**
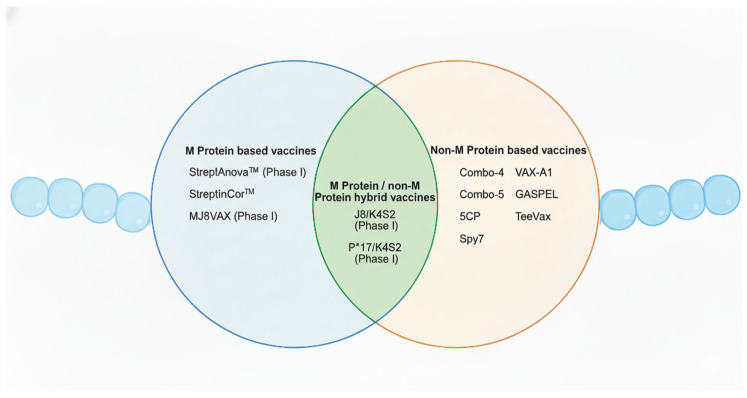
Overview of the Group A Streptococcus vaccine candidates addressed in this review. This Venn diagram summarises GAS vaccine candidates categorised into M protein-based, non-M protein-based, and hybrid vaccine approaches. Candidates that have completed phase I clinical trials are indicated. Created in BioRender. Fan, S. (2026). https://BioRender.com/t0tfrmi.

In recent years, significant progress has been made with the establishment of human infection models. For instance, one study successfully determined a safe dose of GAS by topically applying *emm*75 strains to the oropharynx of human volunteers, demonstrating a controlled and ethical approach to studying infection in humans [133]. This represents a major breakthrough in vaccine research, especially considering that humans are the only natural host of this pathogen. The ability to simulate infection in a safe and controlled human model provides a valuable platform for the evaluation and development of novel vaccines. Currently, GAS vaccine development has entered a more diversified and innovative phase. Beyond the traditional protein subunit vaccines, newer platforms now include peptide/epitope-based vaccines, mRNA vaccines, glycan-conjugated or polysaccharide–protein conjugate vaccines (targeting group A carbohydrate, GAC), and bacterial vector vaccines. For the purposes of this review, GAS vaccines will be categorised based on their antigenic targets into M protein-based and non-M protein-based vaccines. In addition, hybrid vaccines comprising both M protein and non-M protein antigens will also be discussed (Figure 3).

### 4.1. M Protein-Based Vaccine Candidates

The M protein, encoded by the surface *emm* gene, is the principal virulence and immunological determinant of GAS [134]. Consequently, it has been the central focus of most GAS vaccine development efforts. In 1973, the first clinical trial evaluating protective efficacy in humans showed that an M protein vaccine formulated with alum was effective in preventing the upper respiratory tract infection caused by GAS [135]. In light of the antigenic diversity and potential safety concerns, the modern M protein vaccine development has put more emphasis on avoiding cross-reactivity and improving vaccine coverage.

#### 4.1.1. StreptAnova™

StreptAnova™ is a 30-valent vaccine formulation composed of four recombinant fusion proteins containing N-terminal peptide sequences derived from 30 different M protein serotypes. Earlier versions of this vaccine began with a hexavalent recombinant construct comprising six N-terminal serotype fragments, which successfully completed a phase I clinical trial and provided the first evidence of feasibility for recombinant protein-based GAS vaccines in humans [136]. A subsequent 26-valent formulation also demonstrated favourable safety and immunogenicity profiles in human subjects [137]. Considering the epidemiology of pharyngitis and invasive GAS infections, a revised 30-valent formulation was evaluated in rabbits, where it generated broadly opsonising antibodies and suggested even greater protective potential [138]. A phase I clinical trial of the 30-valent StreptAnova™ confirmed its safety and tolerability in humans [139]. More recently, the StreptAnova™ formulation has been adapted into an mRNA–LNP platform. In rabbit models, the mRNA-based vaccine produced robust antibody responses at only approximately one-quarter the dosage of the protein-based vaccine, highlighting the advantages of mRNA technology for rapid multivalent adaptation and antigenic flexibility at lower production costs [140]. However, StreptAnova™ has some major drawbacks. Although designed to cover a broad range of common M types, its formulation is primarily based on strains dominant in high-income countries. In many low-income settings, the circulating strains differ significantly from those in the vaccine, suggesting lower effectiveness compared to North America or Europe [134].

#### 4.1.2. StreptInCor™

StreptInCor™ is another promising candidate that contains 55 synthetic amino acid residues of the C-terminal conserved region of the M protein, including B and T cell epitopes, which are believed to be non-cross-reactive. These epitopes are broadly recognised by diverse HLA class II molecules, suggesting their potential as a universal GAS vaccine [141]. In various murine models, StreptInCor™ has been shown to induce strong immune responses without triggering autoimmune cross-reactivity, and it provided high levels of protection in challenge studies (reviewed in [142]). Furthermore, toxicity studies conducted in minipigs and Wistar rats demonstrated good safety and tolerability profiles, with no significant adverse effects reported [143,144].

#### 4.1.3. MJ8VAX

MJ8VAX, is a minimal epitope vaccine based on a 29-amino acid B cell epitope (J8), which includes a central 12-amino acid sequence derived from the conserved C-repeat region of the M protein. The peptide is conjugated to diphtheria toxoid (DT) as a carrier protein [145]. The use of DT enhances the generation of antigen-specific memory B cells and supports sustained antibody responses. In mouse challenge models, MJ8VAX provided protection against GAS infection (reviewed in [146]). As a representative of peptide-based vaccines, MJ8VAX showed promising safety in preclinical studies and did not induce autoimmune sequelae affecting the heart [147]. A completed phase I clinical trial further confirmed its safety and immunogenicity in human subjects [148].

### 4.2. Non–M Protein Vaccines

Non-M protein antigens have emerged as attractive alternative targets for GAS vaccine development, particularly given the historical concern that immune responses against the M protein may produce cross-reactive antibodies that contribute to autoimmune complications such as ARF and RHD [149]. Due to their higher sequence conservation, non-M protein candidates may provide broader protective coverage than M protein-based vaccines. Candidates have been selected using criteria such as immunogenicity, sequence conservation, surface exposure, and protective efficacy (reviewed in [150]). GAS antigens with protective efficacy in one or more mouse vaccine models have been described in numerous studies and more recent efforts have focused on the development of multicomponent GAS vaccines that offer the promise of broader strain coverage and enhanced efficacy [151,152].

#### 4.2.1. Combo-4

Combo-4, developed by GlaxoSmithKline (GSK), is a four-component subunit vaccine candidate composed of recombinant SpyCEP, SpyAD, SLO, and GAC. Three candidate protective antigens were initially identified using reverse vaccinology approaches [153], and the conjugation strategy between GAC and the carrier protein CRM_197_ was subsequently optimised to improve vaccine design [154]. While GAC–protein conjugates generated detectable anti-GAC titers, their protective efficacy in mouse challenge models remains unclear. Nonetheless, GMP production and toxicological assessment of Combo-4 are underway (reviewed in [21,155]), and it has been acknowledged by the WHO as a competitive candidate in the global GAS vaccine development roadmap [156].

#### 4.2.2. Combo-5

Combo-5 contains five highly conserved GAS antigens, ScpA, SLO, SpyCEP, arginine deiminase (ADI), and trigger factor (TF), formulated with alum as an adjuvant. While it showed protective efficacy in a skin infection model in mice, it failed to provide protection in invasive disease models [157]. In a non-human primate (NHP) pharyngitis model, however, Combo-5 immunisation reduced the severity of pharyngitis and tonsillitis, outcomes that could not be recapitulated in murine models [158]. Importantly, the choice of adjuvant was shown to influence efficacy outcomes. When Combo-5 was formulated with the experimental squalene emulsion-based adjuvant SMQ (containing saponin QS21 and a synthetic TLR4 agonist), protective efficacy against invasive GAS infection greatly improved [159]. A recent study reported a new mRNA–LNP formulation delivering multiple conserved antigens from Combo-5, which protected mice in two invasive infection models by inducing robust B cell and effector T cell responses [160], highlighting the potential of mRNA technology for multicomponent bacterial vaccines.

#### 4.2.3. 5CP

5CP is a protein subunit vaccine consisting of five highly conserved GAS components: SrtA, SLO, SpyCEP, ScpA, and SpyAD. The formulation induced strong target-specific antibody responses and provided protection against mucosal, systemic, and skin infections in murine models [161].

#### 4.2.4. Spy7

Spy7 is a multicomponent subunit vaccine identified using pooled human immunoglobulin recognition. It contains seven highly conserved surface antigens: ScpA, an oligopeptide-binding protein, pullulanase, a nucleoside-binding protein, a hypothetical membrane-associated protein (Spy0762), a cell surface protein (Spy0651), and SpyAD [162]. Immunisation elicited anti-streptococcal antibodies and conferred protection against both M1 and M3 strains of GAS [163]. These findings highlight the feasibility of developing vaccines against extracellular bacteria and support the potential of Spy7 as a conserved non-M protein-based candidate.

#### 4.2.5. VAX-A1

VAX-A1 is a highly effective conjugate vaccine composed of three conserved GAS antigens: SpyAD, SLO, and ScpA. The vaccine employs a SpyAD–GAC^PR^ construct, in which the GAC polysaccharide is de-GlcNAcylated and selectively conjugated to SpyAD, enabling co-delivery of glycan and protein antigens. VAX-A1 conferred significant protection in both systemic and skin infection animal models. Importantly, immune sera showed no detectable cross-reactivity with human myocardial or brain tissue lysates, suggesting improved immunological safety of the GAC^PR^ structure [164]. Additionally, its ScpA component provided cross-protection against Group B Streptococcus [165], further supporting the feasibility of GAC-based conjugate vaccines as a strategy for broad-spectrum streptococcal prevention.

#### 4.2.6. Group A Streptococcus Pilus Expressed on *Lactococcus lactis* (GASPEL)

Group A Streptococcus pilus expressed on *Lactococcus lactis* (GASPEL) is a mucosal vaccine delivered via *L. lactis*, a non-virulent food-grade bacterium expressing seven different GAS pilus proteins. In murine models, combination immunisation elicited systemic antibody responses comparable to single-construct immunisation and induced partial cross-reactivity with non-vaccine strains. The vaccine also conferred protection against a homologous strain in a nasopharyngeal colonisation model [166].

#### 4.2.7. TeeVax

TeeVax targets the pilus major pilus fibre subunit (T antigen) using a recombinant multivalent protein approach. TeeVax1, comprising alternating fusions of six different T antigen domains, induced strong IgG responses in rabbits and showed partial protection against invasive disease in mice [167]. Notably, TeeVax2 and TeeVax3 include expanded T antigen coverage, and immunisation with TeeVax1–3 generated antibodies cross-reactive with over 95% of epidemiologically prevalent T serotypes.

### 4.3. M/Non-M Protein Hybrid Vaccines

Hybrid vaccines, which incorporate both M protein and non-M protein components, aim to combine the high immunogenicity of M protein epitopes with the broader coverage potential of conserved non-M protein antigens. For example, the P*17/K4S2 vaccine combines the P*17 B cell peptide epitope, which is derived from the conserved C3 region of the M protein, with K4S2, an immunogen based on the neutrophil-inhibitory protease SpyCEP [168]. Similarly, J8/K4S2 is another hybrid vaccine combining J8 (a minimal M protein epitope) with K4S2, both of which demonstrated robust immunogenicity in early-stage studies [169], effectively inducing and maintaining protective antibody responses against both respiratory and invasive GAS infections. Notably, the safety of the J8/P*17 combination was also evaluated in a Lewis rat model, showing no evidence of autoimmune sequelae [170]. A recent phase I randomised controlled clinical trial targeting J8/K4S2 and P*17/K4S2 is currently underway [171].

### 4.4. Current Challenges and Future Directions

Despite recent progress, GAS vaccine development still faces several challenges. The lack of standardised animal models and clinical endpoints, particularly those relevant to pharyngeal colonisation and disease, remains a bottleneck. Although NHP models have successfully recapitulated clinical features such as erythema, palatal petechiae, and pharyngeal obstruction [158,172], validated immune correlates are still lacking. Furthermore, antigenic complexity poses a challenge because high genetic variability of targets like the M protein may result in limited coverage and serotype replacement [173]. To stimulate progress, the Strep A Vaccine Global Consortium (SAVAC) was established in 2019 to unite global experts and accelerate vaccine development [21]. SAVAC aims at coordinating research to improve burden-of-disease estimates, strengthening surveillance and laboratory capacity, and boosting advocacy to raise the profile of GAS disease. The SAVAC has also developed a systematic framework to guide research priorities and suggests drawing on lessons from COVID-19 vaccine development, including mRNA platforms and adaptive clinical trial designs. As lead candidates approach clinical testing, the SAVAC is preparing the field by (1) enabling efficacy trials in low- and middle-income countries through better data, surveillance, and trial capacity; (2) engaging vaccine developers to clarify the commercial case and overcome development barriers; and (3) preparing global stakeholders (e.g., WHO, funders, and national policy bodies) to support future implementation.

## 5. Conclusions

Since the discovery of the first GAS virulence factor, M protein, in the early 1930s, a large body of work has led to a much-improved understanding of GAS virulence. Still, novel insight evolves every year, like the identification of novel virulence factors (e.g., CEF and S protein) and the discovery of novel functions of previously characterised proteins, such as the involvement of SpeB in pyroptosis. Many questions still remain, in particular, the involvement of virulence factors in various GAS diseases. GAS has evolved a large number of strains but research has focused mainly on a small subset (e.g., *emm*1, *emm*12, *emm*3, and *emm*28), which dominate in many high-income settings. Even within smaller subsets, virulence often differs. For example, SpeA seems most active in *emm*1- and *emm*3-type strains with less or no activity in other strains. Similar, the DNase Sda1 is virulent mainly in pandemic *emm*1 strains, but redundant or non-essential in many other strains. Importantly, GAS virulence factors are not equally expressed or equally important at all times. Instead, they are part of a hierarchical, context-dependent expression programme shaped by regulatory systems (like CovR/S and others) that respond to host environmental cues, stressors, and metabolic signals. This creates functional dominance of different virulence determinants depending on infection stage and niche rather than mere redundancy (e.g., DNases and complement evasion proteins).

There is currently no GAS vaccine candidate that has proceeded beyond phase II clinical trials. Vaccine licensure is impeded by antigenic diversity, uncertain correlates of protection, and safety concerns. Fundamental research focusing on GAS virulence factors might help mitigate these barriers by identifying conserved, essential targets, defining protective immune mechanisms, and guiding rational antigen combinations and regulatory-relevant efficacy endpoints.

## Data Availability

No new data were created or analyzed in this study. Data sharing is not applicable to this article.
